# Biomarkers and Signaling Pathways Implicated in the Pathogenesis of Idiopathic Multicentric Castleman Disease/Thrombocytopenia, Anasarca, Fever, Reticulin Fibrosis, Renal Insufficiency, and Organomegaly (TAFRO) Syndrome

**DOI:** 10.3390/biomedicines12061141

**Published:** 2024-05-21

**Authors:** Remi Sumiyoshi, Tomohiro Koga, Atsushi Kawakami

**Affiliations:** 1 Department of Immunology and Rheumatology, Division of Advanced Preventive Medical Sciences, Nagasaki University Graduate School of Biomedical Sciences, 1-7-1 Sakamoto, Nagasaki 852-8501, Japan; remis@nagasaki-u.ac.jp (R.S.); atsushik@nagasaki-u.ac.jp (A.K.); 2 Clinical Research Center, Nagasaki University Hospital, Nagasaki 852-8501, Japan

**Keywords:** iMCD-NOS, iMCD-TAFRO, TAFRO syndrome, IL-6, CXCL-13, JAK-STAT3 pathway, PI3K/Akt/mTOR pathway, Type I IFN, PD-1

## Abstract

Idiopathic multicentric Castleman disease (iMCD) and TAFRO syndrome present a variety of symptoms thought to be caused by excessive inflammatory cytokines and chemokines, but the underlying mechanisms are unknown. iMCD is broadly classified into two types: iMCD-NOS and iMCD-TAFRO, which have distinct laboratory findings, pathological features, and responses to treatments. It is thought that iMCD-NOS, particularly the IPL type, responds favorably to IL-6 inhibitors due to its IL-6-centric profile. iMCD-TAFRO frequently progresses acutely and seriously, similar to TAFRO syndrome. Elevated levels of cytokines, including IL-1β, TNF-α, IL-10, and IL-23, as well as chemokines like CXCL13 and CXCL-10 (especially in iMCD-TAFRO), SAA, and VEGF, have been linked to the disease’s pathology. Recent research has identified key signaling pathways including PI3K/Akt/mTOR and JAK-STAT3, as well as those regulated by type I IFN, as crucial in iMCD-TAFRO. These results suggest that dominant pathways may vary between subtypes. Further research into the peripheral blood and lymph nodes is required to determine the disease spectrum of iMCD-NOS/iMCD-TAFRO/TAFRO syndrome.

## 1. Introduction

Castleman disease (CD), initially characterized by Dr. Benjamin Castleman in the mid-20th century [1], manifests in two principal forms: the localized form, unicentric CD (UCD), in which lesions are limited to a single surgically resectable site, and multisite, multicentric Castleman disease (MCD), in which lesions spread to multiple sites [2]. UCD is frequently asymptomatic and clinically unproblematic following surgical resection, whereas MCD is characterized by the presence of multiple lymph node enlargements and symptoms such as fever, weight loss, night sweats, and fatigue. Biological markers indicate ongoing inflammation, such as high C-reactive protein (CRP) and low albumin and hemoglobin levels. Polyclonal hypergammaglobulinemia is a relatively common condition. Wasting and secondary amyloidosis, which are linked to chronic inflammation, can be problematic.

Although human herpesvirus type 8 (HHV8-associated MCD) can cause MCD [2], in Japan, the etiology of most MCD cases remains unidentified, leading to their classification as idiopathic MCD (iMCD). iMCD encompasses individuals who display characteristics of TAFRO syndrome, which is a clinically established concept. In 2010, Takai et al. coined the term “TAFRO syndrome”, delineating it as a systemic inflammatory condition marked by thrombocytopenia, anasarca (widespread edema, pleural effusion, ascites), fever (systemic inflammation), reticulin fibrosis (bone marrow fibrosis, increased megakaryocytes) or renal insufficiency, and organomegaly (enlarged liver, spleen, and lymph nodes) [3]. Individuals diagnosed with TAFRO syndrome typically present with infrequent occurrences of lymph node enlargement, which, if present, are usually only mildly enlarged. Furthermore, they typically do not exhibit hypergammaglobulinemia. Some patients have clinical features of TAFRO syndrome but CD-like histopathological findings in their lymph nodes, a condition known as iMCD-TAFRO. Other types of iMCD are referred to as iMCD-NOS (iMCD—not otherwise specified). Although some consider TAFRO syndrome a subtype of iMCD, the distinction between iMCD-NOS, iMCD-TAFRO, and TAFRO syndrome (TAFRO-without iMCD) is debatable. An attempt has also been made to categorize iMCD-NOS as idiopathic plasmacytic lymphadenopathy (IPL), which is characterized by marked hypergammaglobulinemia and the sheet-like infiltration of polyclonal plasma cells in the lymph nodes, or non-IPL [4]. iMCD-NOS, particularly the IPL type, frequently responds positively to IL-6 inhibitors during the chronic phase. Generally, the IPL type has a favorable prognosis, and a report from China has also shown that IPL type iMCD has a better prognosis compared to other types [5]. In contrast, iMCD-TAFRO and TAFRO syndrome frequently respond poorly to IL-6 inhibitors and have an acute-to-subacute course. The pathological findings also differ between clinical forms, indicating that iMCD is a diverse group of disorders with distinct pathogenesis. Currently, no definitive biomarkers exist to diagnose iMCD or TAFRO syndrome, nor to clearly distinguish between iMCD-NOS, iMCD-TAFRO, and TAFRO syndrome.

## 2. Relevant Sections

### 2.1. Role of IL-6

The causes of iMCD and TAFRO syndrome are unknown, but they are thought to be pathogen etiologies or paraneoplastic phenomena associated with clonal cellular proliferation. There may also be a role for autoimmune or autoinflammatory processes. The disease is characterized by the widespread proliferation of lymphocytes and a significant increase in cytokines, particularly IL-6 [6,7,8]. Studies on lymph node biopsies have shown IL-6 production to be significantly associated with the hyperplasia of germinal centers, indicating its pivotal role in iMCD’s pathophysiology, although the specific mechanisms triggering IL-6 overproduction remain elusive.

The cytokine storm, with IL-6 as a protagonist, precipitates a hyperinflammatory milieu that is characteristic of the disease. Most patients have elevated circulating cytokine levels, which improve with IL-6 inhibition or other immunosuppressive agents [8,9]. Strategies that inhibit IL-6 pathways, using monoclonal antibodies targeting IL-6 or its receptor, have been noted for their effectiveness in symptom mitigation and lymph node size reduction [10,11]. Human IL-6 transgenic mice and IL-6-expressing recombinant retrovirus-infected mice developed iMCD-like syndrome, which was ameliorated by administering anti-IL-6 receptor mAb administration [12,13,14].

IL-6, a pleiotropic inflammatory cytokine, interacts with a variety of cells, including immune cells, hematopoietic progenitors, and cells within the liver and epidermis, among others. It operates through IL-6R and gp130 molecules, employing both classical signaling and trans-signaling modalities. The binding of IL-6 to its receptors activates a cascade of intracellular signals, notably the JAK/STAT, Ras-MAPK, and PI3K/Akt/mTOR pathways, among others [15].

For iMCD patients, the IL-6 inhibitors such as tocilizumab (TCZ) and siltuximab are first-line therapies. These are also recommended in the guidelines [16]. In Japan, TCZ is commonly administered irrespective of disease activity, with 57% of severe cases, 40% of moderate cases, and 42% of mild cases receiving TCZ treatment among patients diagnosed with iMCD [17]. However, the role of IL-6 in TAFRO syndrome is more complex. Although IL-6 is strongly associated with the symptomatology and progression of the IPL subtype of iMCD, its role in TAFRO syndrome pathogenesis is less clear. IL-6 inhibitors, while important in iMCD treatment, have shown variable efficacy in TAFRO syndrome, implying that alternative or additional pathogenic pathways are at work and warrant further investigation.

### 2.2. Cytokines/Chemokines/Proteins Other than IL-6

In placebo-controlled trials of siltuximab [18], a clear pattern emerged indicating higher response rates among patients with elevated serum IL-6 levels. Moreover, research revealed that certain individuals with MCD, exhibiting low serum IL-6 levels before initiating anti-IL-6 therapy did not exhibit a positive response to the treatment [19]. On the other hand, siltuximab was found to be ineffective in some patients with high serum IL-6 levels, while effective in patients with normal or low IL-6 levels [18]. These discoveries indicate that besides IL-6, other cytokines and chemokines also hold substantial importance in the development of iMCD.

An array of inflammatory cytokines, like IL-1β and TNF-α, known to activate IL-6 via NF-κB signaling, have been implicated in iMCD pathophysiology, further reinforcing the complexity of its inflammatory landscape [20,21]. Some reports highlight that targeting IL-1β might benefit patients unresponsive to IL-6 blockade [22].

In a study comparing cytokine profiles among individuals with iMCD-NOS, iMCD-TAFRO, and healthy controls [23], it was observed that those diagnosed with iMCD-TAFRO exhibited notably elevated levels of serum interferon gamma-inducible protein 10kDa (IP-10) and reduced levels of platelet-derived growth factor (PDGF)-AA during flare-ups compared to the other two groups. Moreover, patients with both iMCD-NOS and iMCD-TAFRO demonstrated heightened levels of IL-10, IL-23, and vascular endothelial growth factor-A (VEGF-A). Noteworthy is the strong association found between serum IP-10 levels and the occurrence of iMCD-TAFRO, suggesting a potential role of IP-10 in the pathogenesis of iMCD-TAFRO.

In a subsequent investigation [24], researchers analyzed 1129 proteins from six patients with iMCD (13 plasma samples) during both periods of flare-up and remission. They aimed to identify potential contributors to iMCD development and identified heightened levels of acute phase reactants such as serum amyloid A (SAA), haptoglobin, CRP, non-pancreatic secretory phospholipase A2 (NPS-PLA2), and complement 3b (C3b). Additionally, elevated levels of cytokines or chemokines, including the tissue inhibitor of metalloproteases-1 (TIMP-1), chemokine C-X-C motif ligand 13 (CXCL13), C-C motif ligand (CCL) 23 (CCL23), CCL21, and CCL14, were observed during flare-ups. Notably, among these, NPS-PLA2 exhibited the most significant increase (*p* = 0.017). According to gene set enrichment analyses, the chemokines and complement pathways were the sole pathways significantly enriched. These findings suggest a potential causal link between iMCD and chemokine-driven inflammation, commonly referred to as “chemokine storms”. CXCL13, a chemokine crucial for B-cell migration to the germinal center, was identified as the cytokine with the highest abundance among all patients (log2 fold change = 3.22). Immunohistochemical staining showed a significant increase in CXCL13 expression within the lymph node germinal centers of patients with iMCD compared to controls, resulting in a stromal meshwork pattern. Furthermore, IL-10 and IL-23 were found to be upregulated in iMCD-TAFRO cells, but not as much in iMCD-NOS cells.

In a recent study [25], 1178 proteins were analyzed while comparing 88 patients with iMCD to 42 healthy individuals. The study found 14 proteins that showed a more than twofold increase (11 proteins) or decrease (3 proteins) in patients compared to the healthy control group. The 14 proteins with altered expression included acute phase reactants (NPS-PLA2, SAA), chemokines, cytokine pre-B-cell colony-enhancing factors, interleukin-36 alpha (IL-1F6), CCL21, CXCL13, vascular endothelial growth factor (VEGF), and cytokine receptors and binding proteins (tumor necrosis factor receptor superfamily member EDAR (EDAR), interleukin-5 receptor (IL-5 Ra), and insulin-like growth factor-binding protein 1 (IGFBP-1), among others (carbonic anhydrase 6, creatine kinase-MB (CK-MB), denylosuccinate lyase (PUR8), and immunoglobulin E (IgE)). A proteomic study of six patients with iMCD identified 8 of the 14 differentially represented proteins, with a more than twofold difference between relapse and remission phases [24]. The top three proteins with the most significant differential expression—NPS-PLA2, SAA, and CXCL13—confirmed previous findings. They also compared the serum proteome of another group consisting of 23 patients with iMCD to the expected healthy range. Among the forty proteins analyzed, seven had median levels that were significantly higher than the 97.5th percentile for healthy people, with four of them increasing by more than a factor of two above this threshold. CXCL13, macrophage inflammatory protein-1 alpha (MIP-1α), CCL18, and VEGF, each showed a significant overexpression (false discovery rate < 0.0001).

In a novel study involving immunodeficient mice engrafted with lymph node cells from patients with iMCD-NOS [26], the mice that received iMCD-NOS cells displayed a lethal iMCD-like inflammatory response. In contrast, mice engrafted with cells from non-iMCD patients who lacked inflammatory symptoms served as negative controls. Following cell transplantation, peripheral helper T (Tph) cells proliferated, and there was a significant elevation in human CXCL13 levels in the sera of the mice. The administration of neutralizing antibodies against human CXCL13 suppressed the inflammatory response and increased the survival rates of the recipient mice. These findings suggest that Tph cells and their production of CXCL13 play a significant role in iMCD-NOS, supporting the disease’s classification as an immunoregulatory disorder.

In our investigation, we comprehensively analyzed 507 proteins from four cases of iMCD-NOS and two cases of iMCD-TAFRO. This analysis revealed IGFBP-1 as a common factor among patients who responded well to IL-6 inhibitors [27]. We proceeded to measure serum IGFBP-1 levels in 6 patients with iMCD-TAFRO, 13 with iMCD-NOS, and 28 healthy controls. The results showed significantly higher serum IGFBP-1 levels in patients with both iMCD-TAFRO and iMCD-NOS than healthy controls. In addition, patients with iMCD-TAFRO had significantly higher levels than those with iMCD-NOS [27].

Collectively, several studies have found that CXCL13, VEGF, NPS-PLA2, SAA, and IGFBP-1 are commonly elevated in patients with iMCD, implying that these substances may play a role in iMCD pathogenesis.

## 3. Alternative Perspectives on the Pathogenesis of iMCD

### 3.1. Association of iMCD with Autoinflammatory Diseases

Investigations into the genetic landscape of iMCD have unveiled associations with germline mutations typically implicated in autoinflammatory disorders, such as those seen in familial Mediterranean fever (MEFV). Studies have documented instances where these mutations co-occur with iMCD [28,29,30,31]. Specifically, research has highlighted cases in which mutations in the CECR1 gene, linked to adenosine deaminase 2 deficiency, lead to CD-like symptoms in children. Notably, these symptoms have been shown to be amenable to IL-6 blocking treatments [32,33].

The interconnection between autoinflammatory genes and iMCD was further substantiated by a Japanese study, wherein a subset of patients with iMCD was screened for mutations in genes frequently involved in autoinflammatory conditions. The examination, which applied targeted next-generation sequencing to 31 genes, identified MEFV gene variations in a significant portion of the cohort. Distinguishing these individuals based on the presence of certain MEFV variants, their clinical profiles were scrutinized, revealing that patients with specific MEFV mutations experienced more frequent febrile episodes and exhibited lower hemoglobin levels, compared to those without such variants [34].

Of particular interest was the discovery of a novel Ile729Met mutation in one patient, found within exon 10 of the MEFV gene. Advanced molecular dynamics simulations inferred that this mutation could potentially disrupt the integrity of the pyrin B30.2 domain. Such a disruption might lead to inflammasome activation and subsequent elevation in inflammatory cytokines, providing a mechanistic link between MEFV mutations and enhanced IL-6 synthesis, which could underlie the pathogenesis of iMCD [30].

### 3.2. Exploring the PI3K/Akt/mTOR Cascade

Recent advancements in the treatment of idiopathic multicentric Castleman disease (iMCD) have pointed to the efficacy of sirolimus, an mTOR inhibitor, particularly in cases that have not responded to traditional IL-6 inhibitors [35]. This finding highlights the role of the PI3K/Akt/mTOR signaling pathway in the progression of iMCD. Targeting this pathway may provide dual benefits by both restraining the proliferation of T and B lymphocytes and by downregulating VEGF expression, which is crucial for those with resistance to IL-6-targeted treatments.

Further investigations have shown an enhancement of the PI3K/Akt/mTOR pathway in iMCD, particularly among patients who exhibit resistance to IL-6 inhibitors. Signs of this include heightened activity in CD8-positive T cells and increased VEGF-A serum levels, as detected by flow cytometry and proteomic studies [24,36]. The administration of sirolimus has been documented to temper these activations, thereby facilitating clinical remission [36].

In our research, we utilized RNA sequencing to delve into the regulatory mechanisms of iMCD at the cellular level. We observed upregulation in mTOR-related pathways in CD4-positive T cells of iMCD-TAFRO patients when contrasted with their iMCD-NOS counterparts. Remarkably, in iMCD-NOS patients who were resistant to IL-6 inhibitors, subsequent treatment with sirolimus demonstrated a marked downregulation in the activity of mTOR-related pathways. This includes specific signaling pathways related to mTOR itself, as well as those implicated in Huntington’s disease and the elF4 and p70S6K pathways. These observations provide evidence for an alternative, the IL-6 independent mechanism of disease modulation via mTOR activation in both iMCD-TAFRO and IL-6 inhibitor-resistant iMCD-NOS [37]. In light of these findings, mTOR inhibitors like sirolimus emerge as promising therapeutic options.

### 3.3. JAK-STAT3 Pathway

Despite the benefits of IL-6 inhibition in iMCD management, it may not prove beneficial for all individuals. A comparison between those who did and did not respond to the IL-6 inhibitor siltuximab showed that both groups exhibited pronounced IL-6-JAK-STAT3 signaling, suggesting this pathway’s involvement beyond the direct effects of IL-6 [38]. In vitro studies on the peripheral blood mononuclear cells of iMCD patients in remission revealed an increased IL-6 sensitivity, which could be mitigated by JAK1/2 inhibition, indicating a potential therapeutic avenue [39].

An examination of lymph node tissues from iMCD patients showed a notable increase in pSTAT3 within the interfollicular regions when compared to healthy tissue. Intriguingly, the expression levels of IL-6 or pSTAT3 were comparable between those who responded to siltuximab and those who did not, pointing to the complex nature of signaling in this disease [38]. The consistency of IL-6-JAK-STAT3 signaling across patient responses, the ability to temper cytokine hypersensitivity with JAK1/2 inhibitors, and the increased pSTAT3 expression in affected tissues all implicate the JAK-STAT3 axis as a central component in the pathogenesis of iMCD. This might occur through alternative activators or irregularities in IL-6 signaling. Consequently, alternative intervention points within the IL-6-JAK-STAT3 cascade, including JAK1/2 inhibitors like ruxolitinib, merit consideration for their potential to treat patients who are unresponsive to siltuximab. In fact, there is a case report of pediatric patient refractory to siltuximab and chemotherapies responding to ruxolitinib [40]. In Japan, a clinical trial of filgotinib for iMCD is also ongoing (https://rctportal.niph.go.jp/en/detail?trial_id=jRCT2071230108, accessed on 14 May 2024). Filgotinib is reported to be highly JAK1-selective, causing fewer adverse events in the hematopoietic system, and may be better in the treatment of patients with iMCD with anemia and thrombocytopenia.

### 3.4. The Involvement of Type I IFN as a Contributing Factor

In pursuit of elucidating the cellular processes central to iMCD-TAFRO, a study employed cellular and transcriptomic approaches to pinpoint the potential drivers of this condition [41]. Flow cytometry was used to contrast cellular alterations during iMCD-TAFRO flare-ups against remission periods and healthy controls. A noteworthy finding was the dominance of CD8+ T cells within the CD3+ T cell population in iMCD-TAFRO patients, signaling an activation of these cells. Moreover, a particular increase in CD56bright NK cells relative to CD16+ NK cells was observed during disease flare-ups. There was also an observed inclination towards a higher ratio of classical monocytes to nonclassical monocytes in patients both during and outside of flare-ups, with a surge in the absolute count of classical monocytes evident during exacerbations.

Further investigation using single-cell RNA sequencing (scRNA-seq) on paired samples from iMCD-TAFRO patients aimed to unravel the intricacies of T cell activation and the expansion of innate cells during disease escalation. Through the comparative analysis of gene expression during flare-ups and remission, researchers used Gene Set Enrichment Analysis to discern the prevalence of hallmark gene sets. A significant enrichment of the HALLMARK_INTERFERON_ALPHA_RESPONSE gene set was discovered within clusters of monocytes and NK cells, as well as CD4+ and CD8+ T cells. The elevated expression of these genes was a consistent observation across different immune cell populations and patients during exacerbation phases, highlighting a pronounced IFN-I gene signature.

The study also unveiled an association between the expression of IFN-I response genes and the mTOR gene signature in classical monocytes. Notably, when monocytes and T cells from iMCD-TAFRO patients in remission were stimulated with IFN-I in vitro, an augmented mTOR activation was detected, as compared to healthy individuals. However, this response could be diminished by inhibitors targeting mTORC1 or JAK1/2. These insights strengthen the notion that IFN-I signaling is implicated in iMCD-TAFRO’s pathogenesis by promoting JAK-dependent mTOR activation. This opens up the potential for targeting IFN-I as a therapeutic avenue in iMCD-TAFRO, and it is anticipated that clinical trials exploring IFN-I inhibitors, like anifrolumab, will provide further clarity.

### 3.5. T Cells and PD-1 Involvement

T cells play an important role in the immunopathology of iMCD, a group of disorders distinguished by systemic lymphoproliferation and multiple organ involvement. The activation and regulation of T cell subsets are critical to understanding iMCD’s disease mechanisms, particularly in the TAFRO subtype, which exhibits a distinct set of symptoms such as thrombocytopenia, anasarca, fever, reticulin fibrosis/renal insufficiency, and organomegaly (iMCD-TAFRO). Studies using flow cytometry analysis of peripheral blood have shed light on the dynamics of T cell behavior during the disease’s episodic flare phases, revealing the significant activation of both CD8+ and CD4+ T cells, as indicated by increased activation markers [36,41]. This hyperactive state is accompanied by a concomitant decrease in regulatory T cells (Tregs) [42], which are normally associated with maintaining immune tolerance.

In the context of iMCD-TAFRO, the delicate balance of T cell subsets is further disrupted, as evidenced by a decrease in the relative frequency of CXCR5+CD4+ T cells in peripheral blood during flare-ups. This particular finding suggests an increased migration of CXCR5+ cells to the germinal centers, which is a critical site for B-cell maturation and antibody production, implying a link to the pathogenesis of iMCD-TAFRO. Within the CXCR5+CD4+ T cell population, there appears to be a rise in the frequency of a specialized subset known as circulating follicular helper T cells (cTfh) that co-express PD-1 and TIGIT during flares [41]. The upregulation of these markers could be an adaptive response to the inflammatory environment of iMCD-TAFRO.

Type I interferons, which are key modulators of immune responses, are thought to have significant effects in iMCD-TAFRO and have been linked to increased PD-1 transcription [43]. The PD-1 pathway, best known for its role in inducing T cell exhaustion, has emerged as a potential treatment target. The hypothesis that PD-1 signaling plays a role in the pathophysiology of iMCD-TAFRO is supported by the observed modulation of T cell activity during disease flares. Therefore, modulating this pathway with PD-1 agonistic antibodies may induce T cell exhaustion, dampening the abnormal autoimmune responses observed in iMCD-TAFRO. This approach suggests a novel treatment option that may improve the clinical manifestations of this difficult and complex disease.

## 4. Conclusions

**Figure 1:**
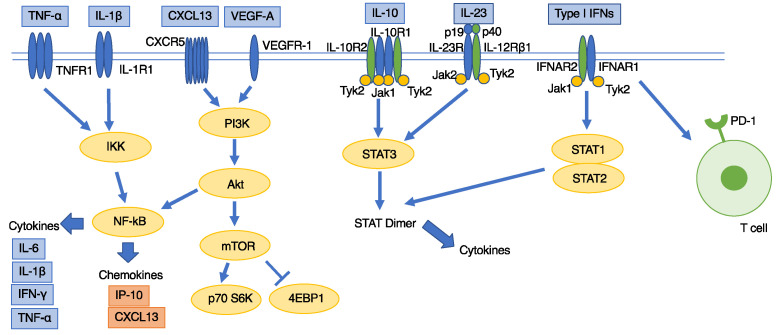
Cytokines/chemokines/proteins that have been reported to be higher in patients with iMCD and their potential activation pathways [44].

Advances in the study of iMCD have shed light on its multifaceted nature, yet it remains a disease characterized by a spectrum of pathogenetic mechanisms, differing between iMCD-NOS and iMCD-TAFRO. Figure 1 illustrates the elevated cytokines, chemokines, and proteins in iMCD, alongside the implicated activation pathways. The common consensus suggests that overproduction of IL-6, alongside other factors, plays a pivotal role in the pathogenesis of both iMCD and TAFRO syndrome. It is hypothesized that iMCD-NOS, particularly those with idiopathic plasmacytic lymphadenopathy (IPL) characteristics, which demonstrate more consistent histopathology, are predominantly influenced by IL-6. In contrast, other forms of iMCD-NOS, as well as iMCD-TAFRO and TAFRO syndrome, likely involve a more intricate interplay of pathogenic factors that extend beyond IL-6.

Histologically, iMCD-TAFRO is often associated with increased vascularization, whereas iMCD-NOS, especially IPL cases, exhibits plasmacytic infiltration. These differing histological patterns are expected to align with distinct biomarker signatures. However, the relationship between histopathological findings and biomarkers is still unclear and needs to be thoroughly studied. Further investigations are essential to delineate the full spectrum and complex interrelationships of iMCD-NOS, iMCD-TAFRO, and TAFRO syndrome.

It is possible that the expression patterns of cytokines, chemokines, and proteins do not always align between peripheral blood and lymph node necessitating validation across both domains. Peripheral blood analysis has distinct advantages due to its accessibility and ability to collect samples pre- and post-treatment, allowing for the study of treatment-induced changes. The evaluation of both peripheral blood and local lymph nodes is critical to understanding the pathogenesis of iMCD. TAFRO syndrome, while less frequently reported than iMCD, has been found to have similar clinical examination results and treatment responses to iMCD-TAFRO. Since evaluating lymph nodes can be challenging in the absence of lymphadenopathy, histopathological findings from renal biopsies and cytokine/chemokine measurements in thoracoabdominal fluid, as well as peripheral blood evaluation, show promise in elucidating the pathogenesis of TAFRO syndrome.

## 5. Future Directions

In terms of future research, this strategy entails isolating peripheral blood mononuclear cells from both patients and healthy people, followed by bulk RNA sequencing and single-cell RNA sequencing. This comprehensive approach seeks to thoroughly examine pathway expression, shedding light on the underlying mechanisms of the iMCD/TAFRO syndrome. The study does not just compare healthy people to patients; it also looks for differences between the various clinical subtypes of iMCD-NOS/iMCD-TAFRO/TAFRO syndrome and different histopathological types of lymph nodes.

Simultaneously, a parallel investigation will include a serum proteomic analysis to further our understanding of the pathophysiology underlying iMCD/TAFRO syndrome. This multi-omics approach provides a comprehensive view of the disease’s molecular landscape, with the potential to identify new biomarkers and therapeutic targets.

Furthermore, patients with iMCD will have their lymph node tissue examined, as well as the cellular components found in body fluids such as pleural and abdominal effusions in patients with iMCD-TAFRO/TAFRO syndrome. Similar analytical techniques will be used in these contexts to investigate differences between various clinical subtypes of the disease, as well as variations across different lymph node histopathological types. Finally, the overarching goal is to identify biomarkers that accurately reflect the complex pathophysiological mechanisms underlying iMCD/TAFRO syndrome, paving the way for more effective diagnostic and therapeutic approaches.
